# Determination of the Labile Iron Pool of Human Lymphocytes using the Fluorescent Probe, CP655

**Published:** 2007-09-17

**Authors:** Yongmin Ma, Zudong Liu, Robert C Hider, Frank Petrat

**Affiliations:** 1 Department of Pharmacy, King’s College London, Franklin-Wilkins Building, 150 Stamford Street, London SE1 9NH, U.K.; 2 Institut für Physiologische Chemie, Universitätsklinikum, Hufelandstr. 55, D-45122 Essen, Germany.

**Keywords:** lymphocytes, intracellular chelatable iron, fluorescence microscopy, fluorescent probe

## Abstract

The present study introduces a method for determining the labile iron pool (LIP) in human lymphocytes. It is measured using the probe CP655, the fluorescence of which is stoichiometrically quenched by the addition of iron. The intracellular CP655 fluorescence in lymphocytes was quenched by increasing intracellular iron concentrations using the highly lipophilic 8-hydroxyquinoline iron complex. Intracellular fluorescence quenching, mediated by the physiological intracellular labile iron, can be recovered on the addition of excess membrane-permeable iron chelator, CP94. The intracellular probe concentration was measured using laser scanning microscopy. An ex situ calibration was performed in a “cytosolic” medium based on the determined intracellular CP655 concentration and probe fluorescence quenching in the presence of iron. The concentration of the LIP of healthy human lymphocytes was determined to be 0.57 ± 0.27 μM. The use of the fluorescent probe CP655 renders it possible to record the time course of iron uptake and iron chelation by CP94 in single intact lymphocytes.

## Introduction

In the normal individual, intracellular iron levels are under extremely tight control. However, when the iron status is changed, such as during periods of regular blood transfusion, iron in excess of functional requirements is present in the “labile iron pool” (LIP) which readily donates iron to ferritin. In principle, this loosely bound iron in living cells is able to redox-cycle between the two common oxidation states, thereby resulting in free radicals such as superoxide and the hydroxyl radical, which can damage biomolecules including DNA and lipids (Halliwell and Gutteridge, 1999). In order to quantify the LIP, a number of methods (Kozlov et al. 1992; Stuhne-Sekalec et al. 1992; Nielsen et al. 1993; Evans and Halliwell, 1994) including fluorescence spectroscopy (Zanninelli et al. 2002; Breuer et al. 1995; Petrat et al. 1999, 2000; Stäubli and Boelsterli, 1998; Loyevsky et al. 1999; Kakhlon et al. 2001; Darbari et al. 2003) have been introduced. Although progress has been made in recent years towards the successful application of fluorescent indicators for the determination of the intracellular labile iron pool in hepatocytes (Petrat et al. 1999, 2000; Stäubli and Boelsterli, 1998) and erythrocytes (Loyevsky et al. 1999; Kakhlon et al. 2001; Darbari et al. 2003), the LIP of lymphocytes has been hardly studied. Lymphocytes are present in the systemic circulation and as such experience continual exposure to relatively high oxygen concentrations leaving the rise of iron-dependent free radical formation. Gackowski et al. 2002 have reported a method to measure the lymphocyte chelatable iron pool based on the fluorescent probe calcein. However, there are several disadvantages associated with this method, namely its lack of selectivity and the requirement to use ester analogues in order to achieve sufficient cellular uptake. We have recently reported the synthesis of several fluorescent probes containing 3-hydroxypyridin-4-one (HPO), which have similar iron-binding properties to that of typical HPOs and contain a fluorescent reporter group that undergoes quenching on interaction with iron (Ma et al. 2004, 2005, 2006), and found 7-diethylamino-N-((5-hydroxy-6-methyl-4-oxo-1,4-dihydropyridin-3-yl)methyl)-N-methyl-2-oxo-*2H*-chromen-3-carboxamide (CP655) to be the most sensitive to the presence of iron. Therefore, in the present work we study the suitability of the HPO fluorescent iron indicator CP655 to selectively chelate and quantify the labile iron pool of isolated human lymphocytes.

## Materials and Methods

### Materials

RPMI-1640 medium, fetal calf serum (FCS), ferrous ammonium sulfate, ferric chloride, Chelex 100, L-ascorbic acid, glutathione, sodium citrate dihydrate, D-(+)-glucose, 8-hydroxyquinoline, poly-L-lysine and propidium iodide were obtained from Sigma-Aldrich (Steinheim, Germany). Ficoll-Paque was from Pharmacia (Piscataway, NJ). Dimethyl sulfoxide (DMSO), potassium chloride, di-sodium hydrogen phosphate dihydrate, magnesium chloride hexahydrate and imidazole were purchased from Merck (Darmstadt, Germany). Falcon cell culture flasks and cell culture-grade Petri dishes came from Becton Dickinson (Heidelberg, Germany) and glass coverslips were from Assistant (Sondheim/Röhn, Germany). Polypropylene tubes for centrifugation came from Nunc (Wiesbaden, Germany). The iron chelator 1, 2-diethy-3-hydroxy-pyridin-4-one (CP94) was synthesized and characterized as reported by Dobbin et al. (1993). The fluorescent probe CP655 was synthesized as previously reported (Ma et al. 2004) and has been identified by nuclear magnetic resonance (NMR) and the purity assessed by elemental analysis.

### Isolation of lymphocytes

Blood (20 ml) from healthy donors (male members of the institute, 25–35 years old) was collected in two 10 ml sodium heparin preservative-free vacutainer tubes, immediately transferred to a 50 ml falcon tube and diluted with an equal volume (20 ml) of RPMI-1640 medium. 10 ml of this diluted fresh whole blood was then layered over 4.5 ml of Ficoll-Paque (FP) and centrifuged (450 × *g*, 25 °C) for 30 min. The supernatant was aspirated down to approximately 1 cm above the Ficoll-Paque layer. The peripheral blood mononuclear cell (PBMC) layer, containing lymphocytes, was transferred to a 15 ml polypropylene tube which contained 10 ml RPMI with 5% FCS (25 °C). Then the cells were centrifuged (15 min at 450 × *g*, 25 °C) and the supernatant aspirated leaving a final volume of 1ml; the lymphocytes were resuspended in 1 ml warm (37 °C) Hanks’ balanced salt solution (HBSS; 137 mM NaCl, 5.4 mM KCl, 1 mM CaCl_2_, 0.5 mM MgCl_2_, 0.4 mM KH_2_PO_4_, 0.4 mM MgSO_4_, 0.3 mM Na_2_HPO_4_, 25 mM Hepes, pH 7.4) and transferred to a 15 ml polypropylene tube. Cells were used for the measurements within 6 h.

### Determination of the intracellular CP655 fluorescence intensity

**Figure 1.**
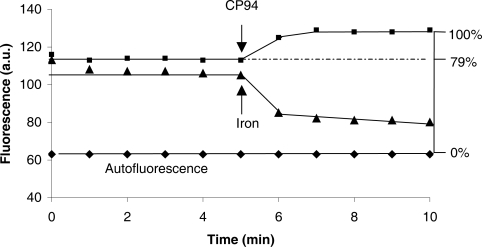
Effect of either iron(III) or CP94 on the intracellular fluorescence of CP655-loaded lymphocytes. Subsequent to the measurement of the initial fluorescence (λ_ex_ = 425± 22.5 nm, λ_em_ = 450–490 nm) of lymphocytes that had been loaded with 30 μM CP655 (for 10 min, in HBSS, 37 °C), either the iron complex (ferric chloride:8-hydroxyquinoline = 15 μM : 30 μM) or the strong iron chelator, CP94 (1 mM), was added to the supernatant and the fluorescence (in arbitrary units, a.u.) was recorded using digital fluorescence microscopy. In addition, the autofluorescence from a parallel incubation of unloaded lymphocytes was recorded at the same instrument settings. The autofluorescence was set at 0% fluorescence and fluorescence after dequenching by CP94 at 100%. Each trace shown is the average of 15–20 cells and is representative of 6 experiments.

Freshly isolated lymphocytes were seeded at a density of 1.5 × 10^7^ cells/ml on a glass coverslip that has been coated with poly-lysine (5 μg/cm^2^) in a modified Pentz chamber. After 10–15 min of incubation at 37 °C, the adherent cells were washed twice with HBSS and loaded with CP655 (30 μM; clogP of iron free ligand at 0.43 and of iron complex at 0.29) for 10 min in HBSS (37 °C). Lymphocytes were then carefully washed three times with HBSS and 6 ml of fresh HBSS (37 °C) remained within the chamber during measurements. The fluorescence measurements were performed using an inverted microscope (Axiovert 135 TV, Zeiss, Oberkochen, Germany) equipped with the Attofluor imaging system (Atto Instruments, Rockville, MD). The CP655-loaded cells were excited at 425 ± 22.5 nm and the emission was monitored at 450–490 nm every 60 s using a bandpass filter. The exciting light was attenuated to 4% using gray filters in order to avoid photochemical damaging of the cells. The intracellular chelatable iron pool was manipulated 5 min after the beginning of the measurements by adding either the membrane-permeable iron(III)-8-hydroxyquinoline complex (15 μM) that had been freshly prepared from stock solutions of ferric chloride (10 mM) and 8-hydroxyquinoline (8-HQ; 20 mM) in dimethyl sulfoxide (DMSO) or by the membrane-permeable iron chelator, CP94 (1 mM; clogP of iron free ligand at 0.16 and of iron complex at −0.40), which had been freshly prepared by dissolving CP94 (100 mM) in 18 mΩ Millipore water. CP655 fluorescence was then recorded until it stabilized (Fig. 1). The autofluorescence from a parallel experiment with unloaded control lymphocytes as obtained using same instrument settings was set at 0% probe fluorescence intensity. The fluorescence intensity of the probe-loaded lymphocytes at the end of the experiment with CP94 was set at 100% and used to calculate the relative initial CP655 fluorescence intensity of the cells (Fig. 1). Since the measurements were performed on the single cell level, it was possible to differentiate lymphocytes from other PBMC, like monocytes. Solely lymphocytes were selected for evaluation.

### Determination of the intracellular probe concentration

Cells were loaded with CP655 as described above. After washing the lymphocytes with HBSS, fluorescence measurements were performed using a laser scanning microscope (LSM 510, Zeiss, Oberkochen, Germany) equipped with an argon laser. The intracellular fluorescence of CP655, after being fully dequenched by CP94 (1 mM), was excited at 458 nm and observed through a 475 nm bandpass filter. The fluorescence measurements were performed in a focal plane 5–10 μm above the surface of the glass cover-slip. At the end of the experimental procedures the uptake of the vital dye propidium iodide was routinely determined in order to detect loss of cell viability. The red fluorescence of propidium iodide excited at 543 nm using the helium/neon laser was collected through a 560 nm long-pass filter; nuclear staining of few dead cells suggested that the lymphocytes studied were not proliferating. This focal plane and identical scanning parameters for cellular CP655 fluorescence were used for scanning the cellular autofluorescence as well as the fluorescence of a Chelex 100-treated tris(hydroxymethyl)amino–methane buffer (Tris-buffer, pH 7.4, 37 °C) containing known concentrations of CP655. The intracellular concentration of CP655 in lymphocytes was determined from the difference in fluorescence of CP655-loaded lymphocytes and unloaded cells (ΔF) compared with the fluorescence of iron-free CP655 standards in the Tris-buffer.

### Ex situ calibration for the determination of human lymphocyte LIP

The quenching effect of Fe(II) on CP655 fluorescence in a medium designed to simulate the composition of the cytosol (37 °C) was determined using digital fluorescence microscopy at the same settings as used for the cellular measurements (see above). This cytosolic medium contained 100 mM KCl, 5 mM Na_2_HPO_4_, 4 mM ATP, 2 mM MgCl_2_, 6.85 mM glucose, 0.138 mM pyruvate, 1.5 mM L-lactate, 0.23 mM sodium citrate, 2.99 mM potassium phosphate, the amino acid composition of Eagle’s minimum essential medium, 2 mM ascorbic acid, 4.5 mM glutathione (GSH) and 10 mM imidazole, pH 7.2. Aliquots (2 ml) of the medium were placed on a glass coverslip within a modified Pentz chamber, and CP655 at a final concentration of 6 μM, i.e. the intracellular concentration of the probe (see below), was added and mixed with the medium. After the initial stable fluorescence had been recorded, known concentrations of Fe(II) were added from a freshly prepared stock solution (0.5 mM ferrous ammonium sulfate plus 10 mM L-ascorbic acid). The addition of iron was continued until no additional fluorescence quenching could be observed.

## Results and Discussion

CP655 fluorescence in lymphocytes could be both decreased and increased by the addition of iron and iron chelators, respectively. The addition of iron(III)-8-hydroxyquinoline (1:2) at a concentration of 15 μM almost completely quenched the intracellular probe fluorescence (Fig. 1). In controls, i.e. no addition of the iron complex, the intracellular probe fluorescence remained stable for at least 30 min. In other experiments, the addition of a large excess of the membrane-permeable iron chelator, CP94 (1 mM, final concentration), significantly enhanced the intracellular probe fluorescence (Fig. 1). The intracellular probe fluorescence in lymphocytes was found to be relatively weak and the probe dequenching to be relatively low when 10 μM probe was used for cellular loading. Therefore, we increased the CP655 loading concentration to 20, 30, 40 and 50 μM, respectively, and found that a maximum fluorescence increase in lymphocytes was obtained when 30 μM of the probe had been used (data not shown). In contrast to low intracellular probe concentrations, which may be insufficient to completely chelate the cellular labile iron, high intracellular probe concentrations may promote probe leakage from the cells during the time-course of the measurements, rendering the system rather insensitive towards the detection of the small pool of intracellular chelatable iron. Thus, 30 μM CP655 was adopted as an optimal loading concentration for the determination of the LIP in lymphocytes. This loading concentration of CP655 did not influence the cell viability as judged by the vital dye, propidium iodide, which was added to the lymphocytes at the end of the experiments.

**Figure 2.**
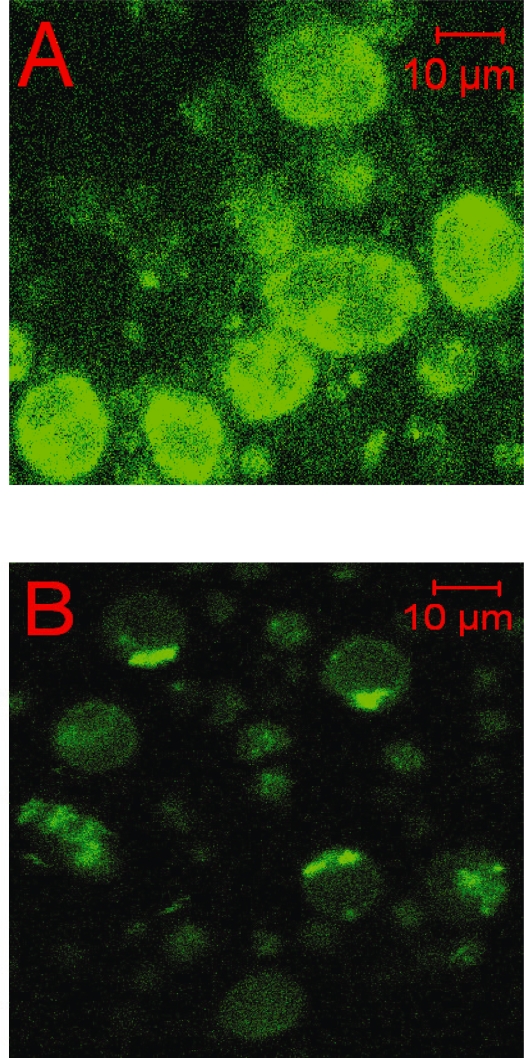
Intracellular distribution of CP655 fluorescence in human lymphocytes. Cells were loaded with CP655 by incubation (30 μM for 10 min in HBSS, 37 °C) and the intracellular fluorescence was scanned using laser scanning microscopy (λ_ex_ =458nm, λ_em_ = 475 nm). (A) Intracellular fluorescence of CP655-loaded cells 10 min after addition of CP94 (1 mM); (B) autofluorescence of unloaded lymphocytes at same instrument settings.

When different CP655 concentrations (0–20 μM, iron-free probe) in a cell-free system were plotted versus their fluorescence intensity as determined using laser scanning microscopy, the fluorescence intensity was found to increase in a linearly proportional fashion with the concentration of the probe standards (data not shown). The total cellular fluorescence (F_total_ = CP655 fluorescence + cellular autofluorescence) was measured 5–10 min after the addition of an excess of CP94 (1 mM) to the supernatant (Fig. 2A). The difference (ΔF) between F_total_ and the autofluorescence of unloaded lymphocytes as determined at the same instrument setting (F_auto_) (Fig. 2B) represented the intracellular CP655 fluorescence (F_CP655_). Based on F_CP655_ and the above standard curve, a mean CP655 concentration of 5.9 ± 1.5 μM was determined in human lymphocytes.

**Figure 3.**
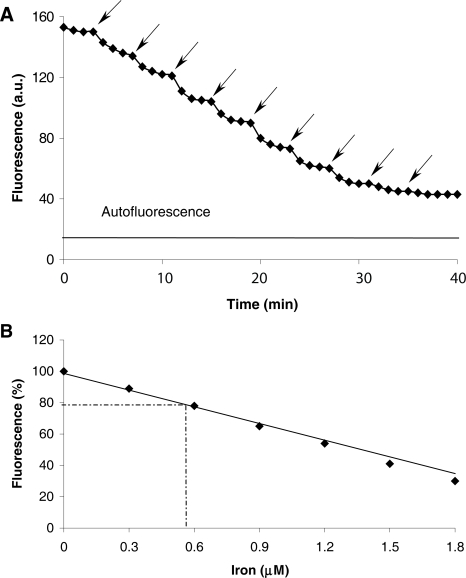
Ex situ calibration of iron(II)-dependent quenching of CP655 fluorescence. (**A**) The fluorescence intensity of CP655 (6 μM) as recorded using digital fluorescence microscopy (λ_ex_ = 425 ± 22.5 nm, λ_em_ = 450–490 nm) in cytosolic medium was quenched by additions of iron (II) (0.3 μM each as shown by arrow) until the progressive quenching leveled to a steady state value. In addition, the fluorescence of the cytosolic medium, without CP655, was recorded at the same instrument settings and used as a blank. Each trace shown is representative for the average of 3 experiments. (**B**) The fluorescence intensity of iron-free CP655 (6 μM) was set at 100% relative fluorescence intensity; 0% fluorescence is equal to the fluorescence of cytosolic medium without CP655. Values shown were obtained from Figure 3A.

CP655 at a concentration of 6 μM, i.e. the intra-cellular probe concentration, was used to perform an ex situ calibration procedure. Calibrations were performed using digital fluorescence microscopy and a cytosolic medium (37 °C) which has been designed to simulate the cytosol composition (Fig. 3A). The fluorescence signal of the iron-free probe (6 μM) was set at 100% probe fluorescence intensity, the autofluorescence of the cytosolic medium in the absence of CP655 was determined at the same instrument settings and set at 0% (Fig. 3B). Small amounts of iron (II) were added to the calibration medium and recordings continued until the progressive quenching of CP655 fluorescence leveled off, suggesting that a steady state had been reached (Fig. 3A). Based on this ex situ calibration curve and relative intracellular fluorescence intensity of the initial CP655 fluorescence of 79 ± 8% (Fig. 1), the concentration of intracellular chelatable iron in lymphocytes was determined to be 0.57 ± 0.27 μM (n = 8). This value is well in line with the value of 0.53 ± 0.58 μM LIP reported for healthy human lymphocytes by Gackowski et al. (2002), and comparable to the levels that have been determined in monocytes (Epsztejn et al. 1997), but much less than the hepatocyte LIP (5.4 ± 1.3 μM) determined in our previous report (Ma et al. 2006). The finding that there is good agreement with the results reported by Gackowski et al. (2002) for lymphocytes, would indicate that, for this type of cell, the use of calcein provides a realistic estimate of LIP.

Previously, we have reported that intracellular ligands for iron like phosphates compete with low CP655 concentrations for iron binding (Ma et al. 2006). However, it in unlikely that these ligands strongly affected the intracellular determination of the lymphocyte LIP as reported here, since the ex situ calibrations were performed in a medium designed to simulate the composition of the cytosol, i.e. in a medium containing physiological concentrations of cellular ligands for iron.

In conclusion, in the present study we introduce an assay for the determination of the LIP in single intact lymphocytes based on a combination of digital fluorescence microscopy and laser scanning microscopy. This assay depends on the specific quenching of CP655 by cellular chelatable iron. The method described herein has the advantages of simplicity, relatively low cost, high sensitivity and permits measurement at the single cell level. CP655, a novel fluorescent iron indicator, has been found to be highly sensitive to the presence of intracellular chelatable iron and does not cause obvious cytotoxic effects. In contrast to calcein (Hasinoff, 2003), the indicator is not prone to oxidative damage in the presence of ferrous labile iron. These properties render it suit-able for monitoring the chelatable iron pool in various cell-types and therefore of providing information on the efficacy of different chelators currently under investigation for treatment of iron overload.
